# How human milk shapes the gut microbiota in preterm infants: potential for optimizing early-life microbial development

**DOI:** 10.20517/mrr.2024.86

**Published:** 2024-12-23

**Authors:** Pamela Thomson, Daniel Garrido

**Affiliations:** ^1^Laboratorio de Microbiología Clínica y Microbioma, Escuela de Medicina Veterinaria, Facultad de Ciencias de la Vida, Universidad Andrés Bello, Santiago 7328000, Chile.; ^2^Department of Chemical and Bioprocess Engineering, Pontificia Universidad Catolica de Chile, Santiago 7328000, Chile.

**Keywords:** Preterm infants, breast milk, gut microbiota

## Abstract

Breast milk plays a crucial role in shaping the gut microbiota of preterm infants, with significant microbial sharing influenced by feeding practices and antibiotics, highlighting the benefits of direct breastfeeding for gut health.

## INTRODUCTION

Preterm very-low-birth-weight (VLBW) infants are at heightened risk of severe health complications due to their physiological underdevelopment, with immediate and long-term consequences^[1,2]^. The diversity of gut microbiota in VLBW infants is initially low but increases over time, with facultative anaerobic bacteria dominating in the early stages^[3]^. Factors such as gastrointestinal immaturity, delayed enteral feeding, limited breast milk intake, antibiotic use, and maternal separation contribute to dysbiosis^[1,4]^. Dysbiosis in early life in VLBW has been associated with sepsis, necrotizing enterocolitis (NEC), and potential long-term effects on physical and behavioral development^[5]^.

Traditionally viewed as a nutritional source, human milk is increasingly recognized for its role in shaping neonatal immune, neurological, metabolic and gut microbiota development^[6]^. Recently, Shama *et al.* addressed an important question: how does the maternal milk microbiota contribute to the assembly of the gut microbial community in VLBW infants, especially during the first months of life^[7]^? This study focused on microbial sharing between mother’s milk and infant stool in 94 mother-infant dyads and 422 milk-stool pairs. The dyads were part of the OptiMoM Fortifier Study (NCT02137473), a multi-center, triple-blind, randomized clinical trial^[8]^. Most infants received antibiotics (92.3%), 2/3 received enteral feeding, and 1/3 pasteurized donor human milk (PDMH) due to insufficient maternal milk volumes. Dose-response relationships between milk bacterial intake and infant gut colonization were quantified, highlighting the impact of feeding practices and antibiotics on these dynamics.

## KEY FINDINGS AND ADVANCES

The study found significant microbial sharing (35%) between mother’s milk and the gut microbiota of VLBW infants^[7]^. This number is similar to breastfed term infants^[9]^. Moreover, breastfed infants had a higher probability of sharing a microbe with their mother, with breastfeeding enhancing microbial sharing compared to other feeding methods. Human milk showed greater microbial diversity than VLBW infant stool samples, correlating with their low gut microbial diversity^[7]^.

The physical process of breastfeeding may expose infants to additional maternal microbes from the skin and milk ducts. The proportion of shared bacteria underscores the pivotal role of mother’s milk not only as a nutritional source but also as a microbial vector shaping gut microbiota. It challenges assumptions that the hospital environment or external factors predominantly shape microbial colonization in VLBW infants. These results advocate for promoting direct breastfeeding whenever feasible, even in the context of neonatal intensive care units (NICUs), to optimize microbial transfer and, potentially, health outcomes.

Daily microbial intakes from milk were strongly associated with the bacterial concentrations observed in infant stool, demonstrating clear dose-response relationships^[7]^. Specific genera in milk, such as *Streptococcus*, were detected in higher concentrations in the gut when administered in larger quantities. This quantitative relationship highlights that microbial colonization in preterm infants is not random but is influenced by the specific bacterial composition and volume of milk intake.

Finally, the relationships between milk microbiota and gut colonization evolved over time and were significantly impacted by feeding practices and antibiotic exposure. Antibiotic exposure disrupted microbial sharing by reducing bacterial diversity, and direct breastfeeding was particularly beneficial in the earlier stages of hospitalization.

These results align with existing research showing that breast milk is a key source of microbial inoculation for the infant gut, particularly for *Bifidobacterium* spp., which dominates the gut microbiota of breastfed infants^[10]^. Previous studies have highlighted how dysbiosis in VLBW infants, driven by factors like antibiotic use and delayed enteral feeding, increases the risk of complications such as sepsis and NEC^[4]^. This study is also important because it quantifies microbial sharing and dose-response relationships, showing that specific bacterial genera in breast milk are associated with gut colonization patterns.

## CHALLENGES AND FUTURE DIRECTIONS

The study also highlights challenges and areas for future research. It is important to further verify vertical transmission beyond 16S rRNA sequencing. Future work should integrate culturomics or metagenomic data at strain level to capture a more holistic view of the maternal-infant microbiome relationships. Moreover, microbial taxa should be considered beyond taxonomy. While taxonomic data provide insights into microbial presence, functional studies are needed to understand how the activity of these microbes influences host health, immunity, and metabolism. Shared microbes belonged to different taxa. Analyzing microbes differentially enriched or depleted by human milk in VLBW infants might provide mechanistic insights into how breast milk shapes the microbiome in these infants.

Another aspect of consideration is the potential influence of delivery type in this intricate colonization process, although some studies argue against a high relevance^[11]^. Further, there could be differences in the chemical and microbial composition of preterm mothers’ milk, compared to term milk, which might influence this process^[12]^. Considering that human milk matures over time since pregnancy and before birth, these differences might shape microbial communities residing in the breast and arriving later at the gut of VLBW infants. Finally, it is up to see if this increased colonization has an actual beneficial impact on the preterm infant in the short or long term.

In addition, these findings could be used to understand antibiotic interference, and even counteract the detrimental effects of antibiotics on microbial sharing in VLBW infants, based on current feeding practices or probiotic supplementation. These findings could also help improve feeding strategies in NICUs, enhancing proper colonization of the gut microbiota in VLBW with clear health outcomes.

This research emphasizes the importance of breastfeeding support, particularly in clinical settings. Policies promoting direct breastfeeding and mitigating unnecessary antibiotic use could have profound implications for improving outcomes in VLBW populations. The discovery of microbial consortia dynamics suggests potential therapeutic avenues, such as engineering milk microbiota or designing microbial consortia for supplementation. These strategies could pave the way for novel interventions targeting neonatal dysbiosis.

Moreover, the observed disruptions caused by antibiotics and the protective potential of breastfeeding are consistent with findings from earlier research advocating for minimal antibiotic exposure and promoting maternal milk use to support healthy gut microbiota development^[13]^. The study’s emphasis on the dynamic nature of microbial interactions provides new insights that could inform personalized feeding strategies to mitigate health risks associated with prematurity.

## CONCLUSION

This study provides compelling evidence that mother’s milk microbiota plays a critical role in shaping the gut microbiota of VLBW infants. By highlighting the significance of microbial sharing, dose-response relationships, and community-level interactions, it advances our understanding of how maternal milk contributes to early-life microbial colonization. The findings underscore the need to prioritize direct breastfeeding and develop targeted strategies to optimize microbial health in preterm infants, with the potential for long-term health benefits.

As we continue to unravel the complexities of the maternal-infant microbiome, this research lays the foundation for novel approaches to neonatal care and microbial therapeutics. This study provides a framework for understanding how mother’s milk can mitigate these disruptions by acting as a natural reservoir of beneficial microbes. It also supports the concept that early microbial interventions could have long-lasting effects on the health trajectory of VLBW infants, particularly in reducing the risk of NEC and sepsis.

## DECLARATIONS

### Copyright

© The Author(s) 2024.
